# A Colonic Organoid Model Challenged with the Large Toxins of *Clostridioides difficile* TcdA and TcdB Exhibit Deregulated Tight Junction Proteins

**DOI:** 10.3390/toxins15110643

**Published:** 2023-11-04

**Authors:** Martina Schneemann, Lucas Heils, Verena Moos, Franziska Weiß, Susanne M. Krug, January Weiner, Dieter Beule, Ralf Gerhard, Jörg-Dieter Schulzke, Roland Bücker

**Affiliations:** 1 Clinical Physiology, Nutritional Medicine, Charité—Universitätsmedizin Berlin, Campus Benjamin Franklin, 12203 Berlin, Germany; 2 Department of Gastroenterology, Infectious Diseases and Rheumatology, Charité—Universitätsmedizin Berlin, Campus Benjamin Franklin, 12203 Berlin, Germany; 3 Core Unit Bioinformatics (CUBI), Berlin Institute of Health at Charité—Universitätsmedizin Berlin, 10117 Berlin, Germany; 4 Institute of Toxicology, Hannover Medical School, 30625 Hannover, Germany

**Keywords:** intestinal epithelial human colonic organoid monolayer, claudin, leak flux diarrhea, leaky gut, super-resolution STED microscopy, tricellulin, channel-forming claudin-2, RNA-seq pathway analysis, cytokines, actin

## Abstract

Background: *Clostridioides difficile* toxins TcdA and TcdB are responsible for diarrhea and colitis. Lack of functional studies in organoid models of the gut prompted us to elucidate the toxin’s effects on epithelial barrier function and the molecular mechanisms for diarrhea and inflammation. Methods: Human adult colon organoids were cultured on membrane inserts. Tight junction (TJ) proteins and actin cytoskeleton were analyzed for expression via Western blotting and via confocal laser-scanning microscopy for subcellular localization. Results: Polarized intestinal organoid monolayers were established from stem cell-containing colon organoids to apply toxins from the apical side and to perform functional measurements in the organoid model. The toxins caused a reduction in transepithelial electrical resistance in human colonic organoid monolayers with sublethal concentrations. Concomitantly, we detected increased paracellular permeability fluorescein and FITC-dextran-4000. Human colonic organoid monolayers exposed to the toxins exhibited redistribution of barrier-forming TJ proteins claudin-1, -4 and tricellulin, whereas channel-forming claudin-2 expression was increased. Perijunctional F-actin cytoskeleton organization was affected. Conclusions: Adult stem cell-derived human colonic organoid monolayers were applicable as a colon infection model for electrophysiological measurements. The TJ changes noted can explain the epithelial barrier dysfunction and diarrhea in patients, as well as increased entry of luminal antigens triggering inflammation.

## 1. Introduction

*Clostridioides difficile* (*C. difficile*) infection (CDI) is the most frequent nosocomial infection of the gastrointestinal tract [1]. *C. difficile* bacteria are Gram-positive, sporulating obligate anaerobes which expand in the human intestinal tract following antibiotic treatment and cause diarrhea and intestinal inflammation. CDI may present with severe bloody diarrhea, e.g., with antibiotic-associated *C. difficile* colitis [2]. Severe progressions of CDI develop pseudomembranous colitis or culminate in the formation of toxic megacolon with possible fatality [3]. Nowadays, the initial therapy for patients with severe CDI is treatment with antibiotics, such as fidaxomicin or vancomycin and metronidazole. In case of refractory and recurrent CDI, a fecal microbiota transplantation with healthy donor stool is increasingly performed as an effective treatment [3,4].

The acute phase of the disease depends on toxinogenic proteins produced by *C. difficile*. Three toxins have been described: The Rho-glucosylating *C. difficile* toxins A (TcdA) and B (TcdB), which are large toxins that are clearly associated with diarrhea and colitis [5,6], and the ADP-ribosylating toxin CDT, which is expressed in hypervirulent strains [7,8]. However, functional studies on the epithelial barrier function together with molecular characterization of tight junction (TJ) protein composition in intestinal cell models and the intestines of infected patients are still lacking. Furthermore, pathomechanisms such as those responsible for TJ alterations and the impairment of epithelial restitution processes in the colon epithelium have not been sufficiently studied.

A general and plausible concept for the disease outcome is that diarrhea and epithelial barrier dysfunction depend on the loss of cells via the direct toxicity of the clostridial toxins and their cell death mechanisms, such as necrosis and apoptosis. However, cell loss was found to play a minor role in CDI during toxin-induced epithelial barrier dysfunction [9,10]. Instead, TJ protein expression and subcellular localization may change after toxin exposure and TJ changes can lead to epithelial barrier dysfunction in the intestine, which is a diarrheal mechanism responsible for leak-flux diarrhea, a passive paracellular flux of water and solutes into the lumen. The intestinal TJs consist of occludin, tricellulin, claudins, and other accessory TJ proteins that maintain barrier function in the intestinal segments. The tightness of the epithelium is determined based on the composition of claudins with barrier-forming or channel-forming properties. Previous studies have identified TcdA and TcdB as inducing changes in F-actin, Zonula occludens protein-1 (ZO-1), and occludin localization in T84 cells [9,11,12]. The TJ changes have been thought to be dependent on cytoskeletal F-actin via glucosylation of Rho GTPases. The toxins TcdA and TcdB target the perijunctional actomysin cytoskeleton first. The integrity of the cytoskeleton is critical for intestinal epithelial barrier function via the regulation of TJs in CDI. Besides the effects on the actin cytoskeleton, the GTPases Rho, Rac, and Cdc42, which are substrate targets of TcdA and TcdB, also regulate cellular events such as cell cycle control [13]. Moreover, they are involved in mitogen-activated protein kinase signaling. It is likely that these toxins have effects on cell physiology that may not involve the actin cytoskeleton. Furthermore, the temporal differences between cell rounding and cell death suggest that other events downstream of actin condensation may contribute to cell death [14]. For example, TcdB alters phospholipase A2 activity in toxin-treated cells, and the data suggest that this process is modulated independently of cytoskeleton-related events [15]. To obtain a more accurate resolution of the relevance of cell death mechanisms and paracellular TJ changes to the barrier-breaking properties of toxins TcdA and TcdB, a cell model that could also be used for electrophysiological measurements had to be developed. We aimed to establish an intestinal organoid model for studying CDI with the advantage of a colonic barrier-specific, cell death-competent, non-immortalized, polarized human cell model that contrasts with standard carcinoma cell lines. Human colonic organoid monolayers were expected to be a more sensitive model for CDI analysis, including the direction of apicobasal effects of toxins and effectors in the gut. Thus, the objective of our present study was to elucidate the pathomechanisms of the epithelial barrier defects in human intestinal epithelial organoid monolayers after exposure to the large clostridial toxins TcdA and TcdB.

## 2. Results

### 2.1. Clostridial Toxins TcdA and TcdB Caused Barrier Dysfunction in Adult Stem Cell-Derived Human Colonic Organoid Monolayers

**Figure 1:**
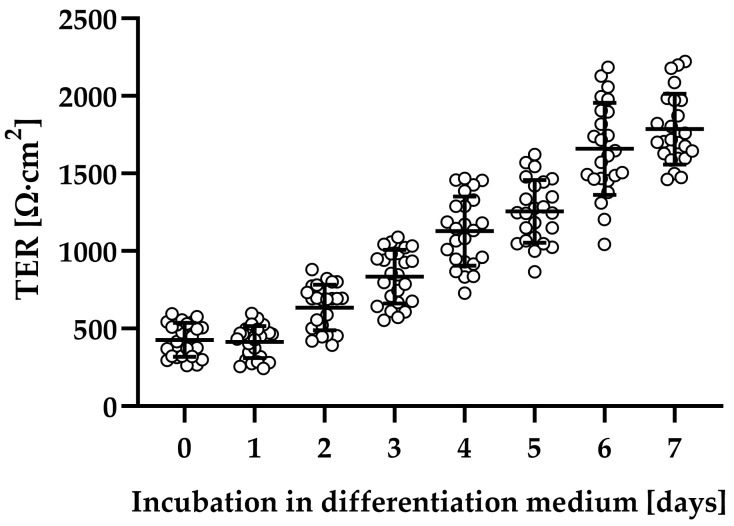
Evaluation of epithelial barrier function of human colonic organoid monolayers. Patient-derived organoids were seeded on filters and grown in expansion medium for two days (TER values not shown). Subsequently, the medium was changed to differentiation medium, and then barrier function was monitored each day by transepithelial electrical resistance (TER) in Ohm∙cm^2^. Human colonic organoid monolayers were used for experiments, usually between 5 and 7 days after application of differentiation medium.

Human colonic organoid monolayers were used to study the effect of *C. difficile* toxins TcdA and TcdB on intestinal barrier function. Patient-derived organoids were successfully established (see Section 4.1), and the adult stem cells were able to form polarized epithelial monolayers with stable transepithelial electrical resistance (TER) after differentiation (Figure 1).

**Figure 2:**
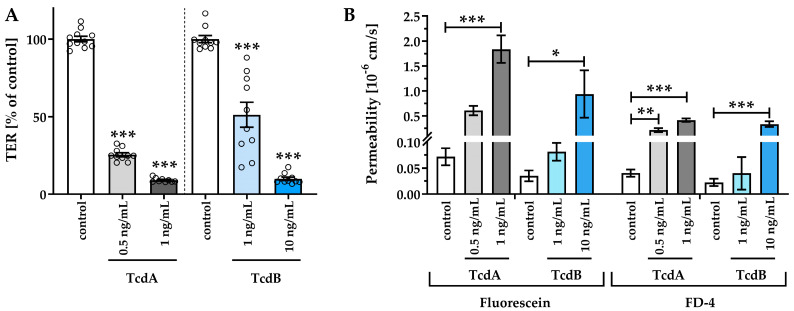
Epithelial barrier function in human colonic organoid monolayers 24 h after exposure to *Clostridioides difficile* toxins TcdA (light gray and dark gray) or TcdB (light blue and dark blue). (**A**) Functional characterization of the effect of TcdA and TcdB on the human colonic organoid monolayers by transepithelial electrical resistance (TER) and (**B**) macromolecular permeability of the paracellular flux markers 332 Da fluorescein and 4 kDa FITC-dextran. TcdA or TcdB was apically applied with a concentration of 0.5 ng/mL, 1 ng/mL or 10 ng/mL. Single datapoints were expressed as circles in (**A**). * *p* < 0.05, ** *p* < 0.01, *** *p* < 0.001, one-way ANOVA, *n* = 5–10.

The human colonic organoid monolayers were treated with 0.5 and 1 ng/mL TcdA, as well as 1 and 10 ng/mL TcdB, respectively. To evaluate epithelial barrier integrity, TER was measured. Although similar concentrations of TcdA and TcdB had different effects on the integrity of monolayers, all toxin-treated human colonic organoid monolayers showed a reduction in TER compared to control human colonic organoid monolayers after 24 h, indicating a barrier defect (Figure 2A). Exposure to TcdB 1 ng/mL and 10 ng/mL reduced TER to 51 ± 8% and 10 ± 1% of controls, whereas TcdA already at a concentration of 0.5 ng/mL and 1 ng/mL led to a TER decrease to 25 ± 1% and 15 ± 6% of the control level. Concomitantly, an increased paracellular permeability towards the fluorescent tracers fluorescein (332 Da) and fluorescein isothiocyanate (FITC)-dextran (4 kDa) was detected (Figure 2B). Permeability for fluorescein in 1 ng/mL TcdA-treated human colonic organoid monolayers was nearly 30 times higher than in control, and 10 ng/mL TcdB was needed to increase the permeability by a factor of 20. Apical-to-basolateral paracellular flux measurements also revealed an increase in permeability for 4 kDa FITC-dextran after 0.5 ng/mL (0.21 ± 0.04·10^−6^ cm/s) and 1 ng/mL TcdA (0.41 ± 0.03·10^−6^ cm/s), as well as after 10 ng/mL TcdB (0.33 ± 0.03·10^−6^ cm/s) exposure each by a factor of 5, 10, or 8 compared to controls (0.04 ± 0.01·10^−6^ cm/s), respectively.

### 2.2. Impaired Barrier Function Emerges before Induction of Necrosis

**Figure 3:**
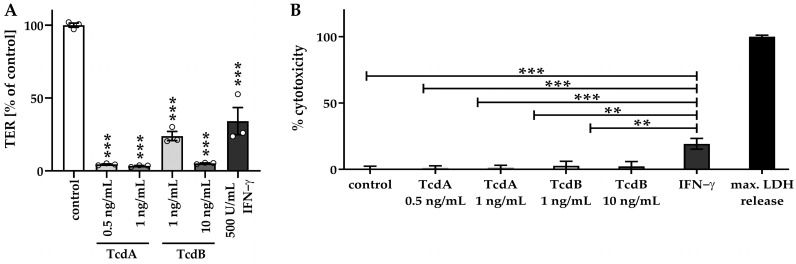
Barrier function in relation to cytotoxicity in human colonic organoid monolayers. (**A**) TER measurement of human colonic organoid monolayers was performed after 24 h of treatment with two concentrations of TcdA (0.5 ng/mL and 1 ng/mL) and TcdB (1 ng/mL and 10 ng/mL) from the apical side, as well as after 72 h of treatment with 500 U/mL interferon gamma (IFN-γ) from the basolateral side. Circles indicate single datapoints. (**B**) To assess necrotic cell death, a lactate dehydrogenase (LDH) release assay was performed. Negative control monolayers remained untreated. Monolayers treated with 500 U/mL IFN-γ were used as a positive control. All values were normalized to the maximum LDH release corresponding to 100% necrosis induction, where monolayers were treated with a detergent lysis buffer. ** *p* < 0.01, *** *p* < 0.001, one-way ANOVA with Bonferroni–Holm correction, *n* = 3–5.

Cell death mechanisms, such as necrosis, can also influence the epithelial barrier function, at least when substantially induced [16], and were evaluated in our organoid model with cytotoxicity assays (Figure 3). Interferon gamma (IFN-γ), which is known to cause necrosis [17], was used as a positive control of cytotoxicity to evaluate the loss of barrier function due to missing cells in the monolayers. With the low-dose toxin treatment, epithelial barrier function was impaired in the human colonic organoid monolayers (Figure 3A). However, no necrosis was induced concomitantly (Figure 3B), as indicated by insignificantly higher cytotoxicity values of 1.1 ± 1.5% in TcdA 0.5 ng/mL, 1.2 ± 1.9% in TcdA 1 ng/mL, 2.7 ± 3.3% in TcdB 1 ng/mL and 2.3 ± 3.6% in TcdB 10 ng/mL treated cells compared to control with a cytotoxicity value of 0 ± 2.4%. In contrast, cells treated with IFN-γ exhibited an increased LDH release of 19.3 ± 4.0%, pointing to the induction of barrier-relevant necrosis (Figure 3B). Thus, these sublethal toxin concentrations were used for the subsequent characterization of barrier defects (Figure 3A).

### 2.3. Down-Regulation of Tight Junction Proteins Claudin-1, Occludin, and Tricellulin via TcdB Caused Disruption of the Epithelial Barrier

**Figure 4:**
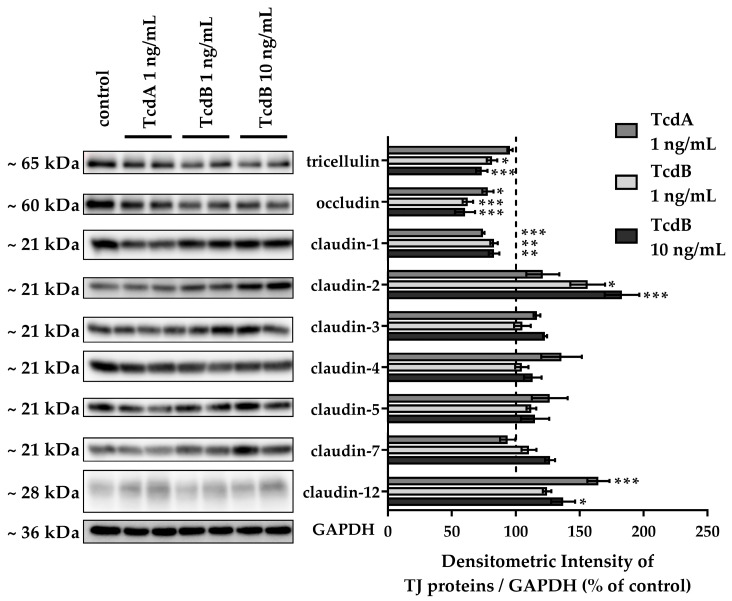
Tight junction protein expression in human colonic organoid monolayers after 24 h of TcdA or TcdB exposure. Representative Western blots are shown with two lanes for each condition. Expression level was analyzed by densitometry on *n* = 4–6 blots, and expression was normalized to the level of glyceraldehyde 3-phosphate dehydrogenase (GAPDH) as loading control. Expression control value is indicated by the dashed line at 100%. * *p* < 0.05, ** *p* < 0.01, *** *p* < 0.001 one-way ANOVA with Bonferroni–Holm correction.

**Figure 5:**
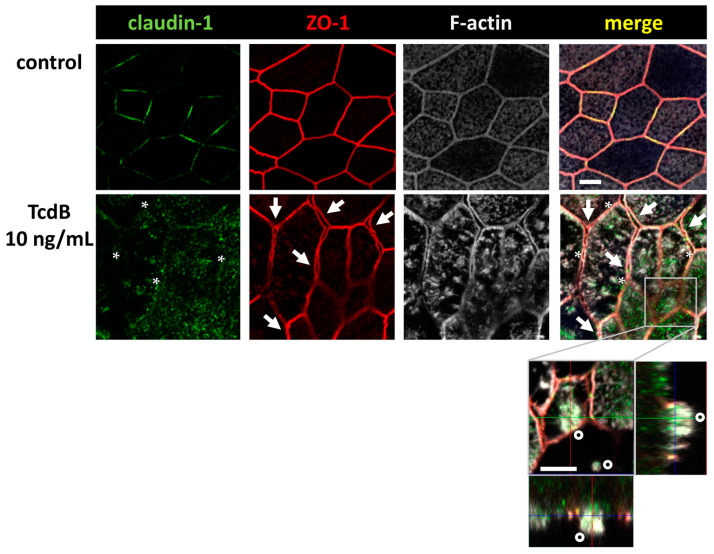
Confocal microscopic images of immunofluorescence staining for claudin-1 (green), ZO-1 (red) and F-actin (white) in human colonic organoid monolayers after 24 h of 10 ng/mL TcdB treatment. F-actin signal appears condensed after TcdB exposure. Asterisks indicate regions with locally reduced claudin-1 signal detected in TJ strands. Arrows indicate signals of ZO-1 being retracted from bicellular TJ connections indicating membranes of neighboring cells begin to split up and separate. Detail orthogonal display of z-stacks shows one cell in a second z-plane with an apical region of highly condensed F-actin indicating a cell which seems to desquamate from the monolayer (circles); bar = 5 µm.

To assess TJ alterations, Western blotting and confocal laser-scanning microscopy (CLSM) after immunofluorescence staining were performed. TJ protein expression in densitometric analysis revealed changes in claudin-1, claudin-2, claudin-12, occludin, and tricellulin after 24 h treatment with either TcdA or TcdB (Figure 4). Claudin-1 and occludin were down-regulated after either TcdA or TcdB exposure. Tricellulin protein expression was down-regulated after TcdB exposure only. The channel-forming TJ proteins claudin-2 and claudin-12 were up-regulated after TcdB exposure, whereas TcdA showed induction only of claudin-12 expression (Figure 4). Claudin-3, -5 and -7 seemed to be unaffected by either TcdA or TcdB. CLSM images reflect the down-regulation of the tightening TJ protein claudin-1 (Figure 5).

We continued our analysis, focusing on TcdB, as it showed clearer microscopic images of our organoid model. In Figure 5, the ZO-1 signal seemed to be affected, too. It appeared that ZO-1 was retracted from the TJ strands, implying a destabilization of the bicellular TJ and the beginning of a separation of neighboring cells (arrows). Concomitant condensation of F-actin (asterisks) and the accompanying collapse of the cytoskeleton may be the reason for the split-up. This in turn destabilized the whole monolayer and contributed to the loss of barrier function.

Furthermore, in contrast to control monolayers, TcdB-treated monolayers showed cells detaching from the monolayer, thus further destabilizing the intestinal barrier. Claudin-1 and occludin down-regulation and ZO-1 and claudin-1 redistribution off the TJ domain can at least in part explain the decrease in TER and the increase in fluorescein flux, pointing to an opening of the leak pathway.

### 2.4. In TJ Domain Integrated Claudin-2 as Defense Reaction towards TcdB

**Figure 6:**
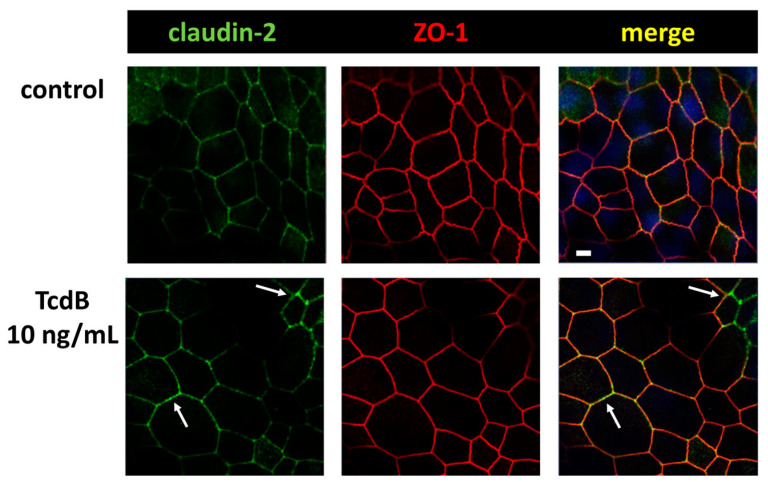
Confocal microscopic images of immunofluorescence staining for claudin-2 (green) and ZO-1 (red) in human colonic organoid monolayers after 24 h of TcdB treatment. Arrows indicate regions with locally increased claudin-2 signal, indicating an increased level of claudin-2 channels in the TJ; bar = 5 µm.

In contrast to the down-regulated expression of barrier-maintaining TJ proteins such as claudin-1, we measured an up-regulation of the channel-forming TJ protein claudin-2 (Figure 4). Moreover, the claudin-2 proteins were integrated into the TJ domain of the epithelial cells, indicating a functional role (Figure 6, arrows). This was remarkable since the pathology of TcdB should force the host cell to disintegrate TJ proteins rather than to promote their assembly into the membrane. Nevertheless, an incorporation of claudin-2 as seen here seems to be possible due to self-organization phenomena and condensation along the functional accessory ZO-1 at bicellular TJs. In our CLSM images from immunofluorescence stainings of claudin-2 in TcdB-treated human colonic organoid monolayers after 24 h, an enhanced claudin-2 signal was observed and more TJs were positive for claudin-2 co-localized together with ZO-1, which points to a functionally active localization of claudin-2 proteins in the TJ domain. Increased numbers of claudin-2 channels in the TJ might explain at least in part the decrease in TER, indicating an opening of the pore pathway (Figure 2A).

### 2.5. Cytokine Measurements in Toxin-Exposed Monolayers

FACS analysis of the organoid cells revealed that the cytokines IL-1β and IL-13 were increased in human colonic organoid monolayers exposed to 10 ng/mL TcdB (1.9-fold of control for IL-1β, *p* < 0.05 and 1.7-fold induction of IL-13, *p* < 0.01, *n* = 5 each).

Directly related to this, it was computed in an Ingenuity Pathway Analysis (IPA) by an RNA-seq dataset of human intestinal HT-29/B6-GR/MR epithelial cell monolayers exposed to TcdA (Table S1) or TcdB (Table S2) that the cytokine pathway IL-1β was clearly activated. In this IPA approach, it was possible to analyze differential gene expression data with upstream regulator analysis providing information on reasons (such as up/down-regulation of cytokine expression) for up- or down-regulation of a particular group of genes. This type of bioinformatic prediction on possible activators or inhibitors (upstream regulators) of a differentially regulated gene set can provide a new hypothesis regarding activation patterns that may have influence in the experimental condition as well as on further cell structures such as TJs. In this upstream regulator analysis, both TcdA and TcdB induced IL-1β as an upstream regulator, with activation z-scores of 2.96 and 2.10 and overlap *p*-values of 8.62 × 10^−4^ and 7.12 × 10^−2^, respectively. The same was true for downstream targets of the IL-13 pathway in TcdB-treated cells, with an activation z-score of 1.50 and an overlap *p*-value of 8.68 × 10^−5^. Furthermore, other cytokine pathways with barrier-affecting properties were activated, such as TNF-α, IFN-γ, IL-4, and IL-22, and further cytokines, such as IL-2, IL-5 or IL-15, were also detected as upstream regulators (Tables S1 and S2).

Additional analysis of our RNA-seq data using gene set enrichment analysis, which is a computer-assisted method that determines whether a gene set has differences between two biological states, revealed activation of inflammatory pathways, such as “TNF-α Signaling Via NF-κB” or “Inflammatory Response” (Figure S1). In addition, both clostridial toxins had a direct effect on the gene sets for “Actin Cytoskeleton Reorganization and Apical Junctions” among others (Figure S1).

### 2.6. Tricellular Tight Junction Protein Delocalization in TcdB-Treated Epithelial Cells

**Figure 7:**
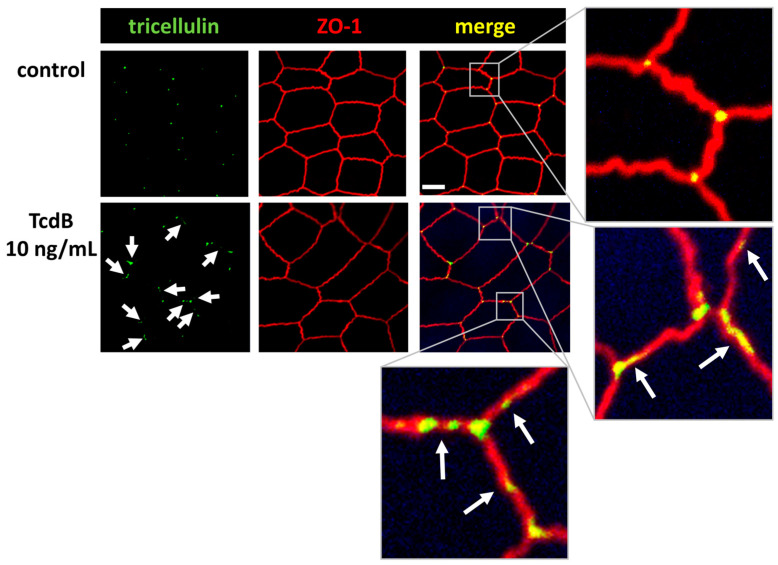
Confocal laser-scanning microscopic images of TcdB-treated human colonic organoid monolayers reveals tricellulin redistribution. Subcellular localization of tricellulin (green) and ZO-1 (red) via immunofluorescence staining. White arrows indicate tricellulin redistribution from tricellular to bicellular TJs; bar = 5 µm.

Further CLSM studies revealed that tricellulin was redistributed from its tricellular TJ membrane domain towards the bicellular TJ domain (Figure 7). This shift may further contribute to the loss of epithelial barrier function towards macromolecules, allowing antigens and pathogens to pass the epithelium and causing mucosal inflammatory responses.

### 2.7. Conventional Laser-Scanning Microscopy and Super-Resolution Microscopy Revealed Subcellular Redistribution of Tight Junction Proteins after Exposure to Clostridial Toxins

**Figure 8:**
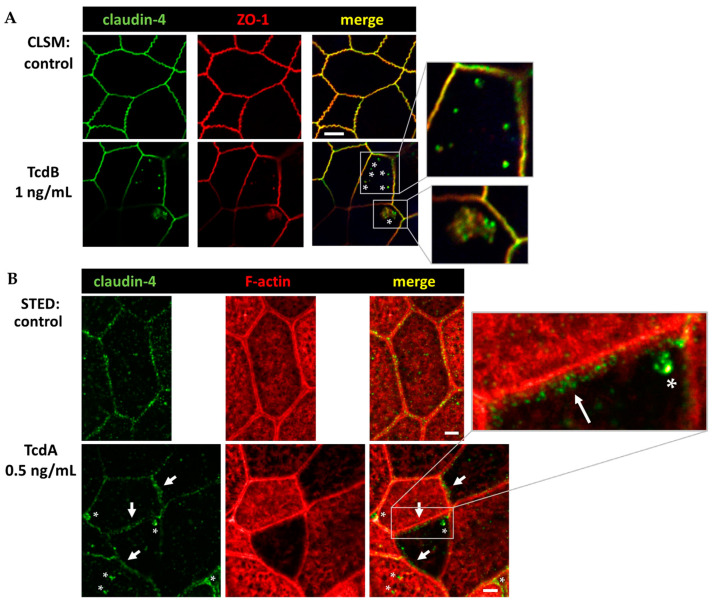
Conventional confocal microscopy and super-resolution STED microscopy of human colonic organoid monolayers. (**A**) Claudin-4 (green) and Zonula occludens protein-1 (ZO-1, red) visualized via immunofluorescence and conventional LSM. Bar = 5 µm. (**B**) Claudin-4 (green) and F-actin (red) protein signals in STED microscopy. Clostridial toxins (TcdA, TcdB) were each incubated with the human colonic organoid monolayers for 24 h in comparable dosages, as given within the Figure. As control, untreated human colonic organoid monolayers were used. White asterisks mark redistributed claudin-4 protein signal into intracellular compartments forming particle-like structures. White arrows indicate claudin-4 accumulation in proximity to the tight junction domain of the membrane. Bar = 2 µm.

**Figure 9:**
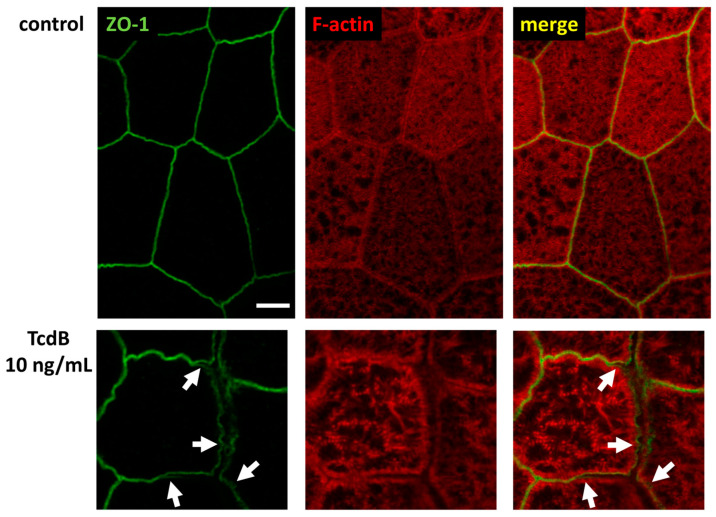
Super-resolution STED microscopy of human colonic organoid monolayers. Microscopic signals of Zonula occludens protein-1 (ZO-1) are shown in green via immunofluorescent staining. F-actin is shown in red via fluorescent phalloidin staining. Upper row: untreated control. Lower row: TcdB-treated cells 24 h after exposure to 10 ng/mL toxin. White arrows indicate delocalization of ZO-1 from bicellular and tricellular contacts. Bar = 2 µm.

In a series of experiments which used immunofluorescence staining and were optimized for conventional laser-scanning microscopy (LSM) or super-resolution stimulated emission depletion (STED) microscopy, the intracellular localization of claudin-4 exhibited obvious changes. A redistribution of claudin-4 protein off the bicellular TJ domain towards small particle-like accumulations in intracellular compartments was visualized via conventional LSM (Figure 8A). This points to a direct molecular correlate of the functional barrier defect, measured after toxin treatment. As an abundant barrier-forming claudin in the large intestine and intestinal cell cultures, claudin-4 was analyzed in more detail with super-resolution STED microscopy (Figure 8B). In STED microscopy (Figure 8B), the TJs in control cells showed a regular shape, while in toxin-treated monolayers, particle-like claudin-4 redistribution was observed, as already seen in Figure 8A. Moreover, in STED micrographs, a fine accumulation of claudin-4 appeared in toxin-treated cells in proximity to the bicellular TJ domain. The cytoskeletal F-actin structure was smooth and without any discontinuities or ruffling in control cells (Figure 8B). In the toxin-treated cells, the perijunctional F-actin appeared to be without any obvious changes, reflecting an intact cytoskeletal organization (Figure 8B). As a sporadic finding of intense toxin activity on the F-actin cytoskeleton, cell detachment in bicellular contacts as well as in tricellular contacts was observed, while the TJ signal represented by ZO-1 was reduced and delocalized in some areas (Figure 9).

## 3. Discussion

To study the major *C. difficile* toxins TcdA and TcdB, at least one of which is present in all CDI, in our organoid model turned out to be rewarding in terms of the different mechanisms leading to barrier dysfunction, such as cell death and opening of different paracellular pathways. As TJ proteins are located at the most apical part of the lateral cell membrane in the intestinal epithelium, they allow tight barriers against the intestinal lumen to form. In CDI, the *C. difficile* bacteria and especially their toxins target the epithelial barrier in the large intestine. To deal with pathomechanisms that more closely resemble disease progression in humans, establishing a new cell model was necessary. The advantages of organoids compared to immortalized cell lines are that organoids exhibit a multicellular composition, higher responsiveness to mediators or noxious agents, higher capability in defense mechanisms, normal healing or repair processes, and epithelial structural maintenance, as well as a higher susceptibility to cell death signals [18,19,20,21].

As a first finding of this study, TcdA was found to be comparably efficient to TcdB at an equimolar low concentration regarding epithelial barrier function in human colonic organoid monolayers. In hamster infection models and colon cell cultures, TcdA was often identified as more potent than TcdB in intoxication experiments [22,23]. However, strains expressing only TcdB are also pathogenic and are found in patients with active CDI, namely when the bacterial load/toxin concentration is just high enough or when such strains have more antibiotic resistance [24,25]. Interestingly, an in vivo study investigating TcdA- or TcdB-deficient *C. difficile* strains, TcdB was found to be more pathogenic compared to TcdA [26]. Thus, taken together, this creates a variability regarding both toxins and the individual importance of the relative toxic extent depending on the cell models and the parameter under investigation.

In our established organoid model [21,27], barrier function was affected by TcdA and TcdB in a complex manner that reflects the situations in colitis, which is also known from other diseases such as ulcerative colitis or Crohn’s disease [28,29,30]. For example, in the colon of ulcerative colitis patients, the expression of channel-forming claudin-2 is often up-regulated, whereas other barrier-maintaining claudins show reduced expression levels, both contributing to a leak-flux type of diarrhea [29,30]. In our study, the impairment of TJ barrier-formers by clostridial toxins is capable of inducing leak-flux, as shown by functional measurements in the organoids. The TER measurements as well as the permeability measurements from apical-to-basolateral direction in the human colonic organoid monolayers showed an increased paracellular flux for small macromolecules. This not only provides evidence for the leak-flux mechanism of diarrhea, but also for the leaky gut phenomenon, explaining mucosal inflammation. Both mechanisms work together and provide one explanation for the inflammatory diarrhea developing in CDI. Barrier dysfunction by TJ deregulation or induction of epithelial cell death may induce a leaky gut, which describes the paracellular uptake of luminal noxes and antigens with the consequence of an immune-mediated self-enforcing barrier dysfunction [31]. In the leaky gut, mucosal cytokines that are released from subepithelial immune cells exhibit barrier-weakening effects, which mediate the ongoing TJ deregulation, thereby down-regulating or redistributing barrier-maintaining TJ proteins such as claudin-1 or -4 or tricellulin. This represents the molecular correlate for the opening of the leak pathway.

Conversely, in human colonic organoid monolayers under the exposure to TcdA or TcdB the channel-forming TJ protein claudin-2 was up-regulated and functionally assembled into the TJ domain of the enterocytes, whereas other claudins were retracted from the TJ. Both, however, can contribute to lowered TER. By induction and functional assembly of channel formers such as claudin-2 and-12, the pore pathway was opened in our human colonic organoid monolayers. Claudin-2 and -12 are described to play a role in calcium homeostasis in the kidney and in the gut [32,33]. The description of claudin-2 as cation and water channel has already been demonstrated [34,35]. As an impressive image of this type of channel formation in the TJ of the colon mucosa, one could imagine that water flows from the tissue through the pores of claudin-2 and -12 into the intestinal lumen like “tears of the gut”, which may have the function of clearing noxious agents from the colonic mucus layer. This kind of defense mechanism against a microbial attack is also conceivably due to the activation of chloride secretion. Despite a few studies reporting claudin-2 up-regulation and bacterial reduction from the mucosa [36], this deserves further exploration. Besides the interesting rinsing off the mucus layers by an up-regulation of channel-forming TJ proteins, the assembly of these proteins in the membrane deserves attention, too. Since other TJ proteins were rather retracted from the TJ domain of the enterocytes in an actin cytoskeleton-dependent manner, it seems questionable how other proteins like claudin-2 were able to assemble in the membrane. One possible explanation for claudin-2 assembly would be the mechanistic view of a self-organized condensation of TJs, the so-called wetting of TJs, which has recently been described for ZO-1 [37]. This kind of assembly of claudins via molecular condensation, depending on their interaction partners in the TJ, could explain the functional induction of claudin-2, while other claudins were disassembled, in our experiments. However, this also requires further investigation with respect to the molecular dynamics of TJ organization. Furthermore, the role of cytokines in regulating TJs should also be further investigated.

Along the leaky gut concept, different cytokines released from the subepithelium can induce distinct deregulation of TJ proteins in epithelial cells. As a prominent example, TNF-α is capable of inducing claudin-2 expression and apoptotic cell death, the latter of which is induced after prolonged incubation with higher doses of TNF-α [38]. Moreover, IL-13 was shown to not only induce claudin-2 but to also reduce tricellulin expression, resulting in enhanced macromolecule permeability [39].

Since most organoids are more susceptible to inflammatory mediators, it is possible that the low concentrations of cytokines such as IL-13 and IL-1β in our experiments have a significant part in TJ effects. These effects are often based on protein expression regulation from the gene, which depends on activated cytokine signaling pathways. Furthermore, the cytoskeletal dysregulation caused by the large clostridial toxins can mechanistically influence TJ proteins, e.g., claudin-4 localization via F-actin depolymerization [40]. Additionally, the low-dose exposure of clostridial toxins used in this study directly influenced the pathways of “Epithelial Structure Maintenance”, “Epithelial Cell Differentiation”, and “Maintenance Of Gastrointestinal Epithelium” as predicted by bioinformatics (Figure S1). Together with the activated “Cytokine Mediated Signaling Pathways in Epithelial Cells”, the observed barrier disruption could be explained at least in part by cytokine induction and in part by cell structure changes such as the cytoskeleton. The barrier-affecting cytokines IFN-γ, TNF-α IL-13, and IL-1β, that were predicted by IPA to be activated upstream regulators or those with positive activation z-scores (Tables S1 and S2) are known as key players in the inflammation of the colon with reduced barrier integrity in ulcerative colitis or *Campylobacter jejuni* infection of the colon [29,41]. This corresponds to the barrier-affecting cytokines and the barrier disruption measured here after exposure of clostridial toxins. Furthermore, in conjunction with cytoskeletal changes, we could sporadically observe reduction of ZO-1 and detachment of cells. This points to focal toxin action with higher impact on F-actin and the TJs due to a focally higher susceptibility or pre-damage or locally accumulated toxin concentrations.

Another recent study confirmed our observations of a barrier defect after exposure to TcdA or TcdB [42]. In this study, the redistribution of ZO-1 within the monolayer was held solely responsible for the measured barrier dysfunction. Interestingly, gene expression of barrier-forming TJ proteins allowing for the formation of a strong and tight epithelial barrier, such as claudin-3 and -4 and occludin was up-regulated, which contradicts the measured TER reduction. Additionally, the toxin concentrations of TcdA and TcdB used in previous studies were much higher compared to the present study [9,11,12,42]. The sublethal concentrations we used probably allowed us to observe more specific effects on TJ proteins and their redistribution away from or to the TJ domain of the cell in the early stage of infection or exposure to the large toxins.

Taken all together, the large toxins of *C. difficile* showed defined effects on the TJ proteins at sublethal concentrations in our organoid model, providing an explanation for the diarrheal mechanism in the early stage and for the cause of mucosal inflammation in CDI patients by barrier dysfunction and the leaky gut phenomenon in the late stage of infection, respectively. The perspective studies that are suitable to prove this leaky gut concept will require animal infection experiments, which are currently being prepared in our lab.

In addition to the large toxins, hypervirulent *C. difficile* strains secrete a binary ADP-ribosylating toxin (CDT) that impairs the actin cytoskeleton and TJ proteins such as occludin, tricellulin, and claudin-4 as a common representative of the claudin family in intestinal epithelial cells [10,43,44]. CDT-positive CDI patients are more likely to have worse clinical outcomes. Therefore, strains that express CDT in addition to the large clostridial toxins lead to increased morbidity and recurrence of CDI, as well as higher mortality rates [45,46]. In our recent study on CDT action in HT-29/B6 cells, the activation of the myosin light-chain kinase (MLCK) [47] was found as one signaling pathway of TJ deregulation [10]. The MLCK induces MLC phosphorylation, which leads to constriction of the perijunctional actomyosin cytoskeleton with the consequence of a redistribution of TJ proteins out of the TJ domain of the cell, including ZO-1, claudins, occludin and tricellulin.

Furthermore, CDT may act like a door opener for the large clostridial toxins [10]. We recently hypothesized that in hypervirulent strains the action of CDT allows the TcdA and TcdB access to the basal compartment of the epithelium. Thus, the large clostridial toxins could then bind to the mainly basolaterally located receptors, such as Frizzled and CSPG4, resulting in a stronger toxigenic potential of e.g., TcdB binding to Frizzled [48,49]. In contrast to the large toxins, with their basolateral receptors, the receptor for CDT is LSR (lipolysis-stimulated lipoprotein receptor, syn. angulin-1), a receptor which is apically located at the tricellular TJ of the enterocytes [50,51]. The mutual opening of the epithelial barrier by toxins with different receptor localizations might be a novel explanation for synergistic toxin effects and the pronounced severity of infection caused by hypervirulent stains. However, also this theory requires further experimental support.

In addition, the immunological aspects of the leaky gut concept for the chronification of colitis needs to be elucidated in additional cellular models, such as a co-culture model of immune cells and epithelial cells, as well as in animal models that may also be considered for the investigation of barrier-stabilizing therapies in CDI.

## 4. Methods

### 4.1. Generation of Intestinal Epithelial Organoids and Organoid Monolayers

Human colonic organoid monolayers were established from patients’ colonic tissue as previously described [21,27]. The isolation and establishment of organoids from the human specimen were approved by “The ethics committee of the Charité”, Berlin (#EA4/015/13). In short, colonic crypts were isolated from healthy human biopsies, put in a Basic Membrane Extract (BME, Cultrex Type II, R&D Systems, Minneapolis, MN, USA) dome, and cultured at 37 °C in a humidified 5% CO_2_ atmosphere in three-dimensional (3D) organoid medium supplemented with stem cell niche specific factors, such as small molecules and inhibitors. This 3D extracellular matrix environment allowed for the formation of patient-derived intestinal epithelial organoids. Medium was changed every 2–3 days. After 7 days, 3D organoids were split into single cells by incubating harvested organoids in TrypLE Express (Gibco, ThermoFisher, Rockford, IL, USA) for 5 min at 37 °C and seeded on BME-coated (1:50 in Advanced DMEM/F12 (Gibco)) polycarbonate filter supports (Millicell-PCF, effective membrane area 0.6 cm^2^, pore size 0.4 µm, Merck Millipore ltd., Darmstadt, Germany). Filters were grown first in expansion medium for two days and afterwards in differentiation medium as described previously [27]. Epithelial human colonic organoid monolayers were used for experiments after reaching confluency and a TER around 1500 Ω·cm^2^, normally between 5 and 7 days after applying differentiation medium. To eliminate any inhibitory effects from medium components, the medium was changed to basic medium AD+++ 24 h prior to toxin or cytokine treatment.

### 4.2. Treatment of Cells and Studies on Epithelial Barrier Function

TcdA and TcdB from *C. difficile* strain VPI10463 were purchased from tgcBIOMICS GmbH, Bingen, Germany. At a period of 24 h prior to toxin treatment, the medium was changed to an inhibitor-free basic medium AD+++ (see Section 4.1). For characterization of intestinal barrier function transepithelial electrical resistance (TER) was measured with a chop-stick electrode pair and a Volt–Ohm meter (EVOM^3^; World Precision Instruments, Sarasota, FL, USA) under sterile conditions after the treatment with different toxin concentrations (TcdA: 0.5 and 1 ng/mL corresponding to 1.62 and 3.25 pmol/L; TcdB 1 and 10 ng/mL corresponding to 3.74 and 37.04 pmol/L). In parallel, epithelial barrier function was further characterized in permeability assays for small macromolecules to test for putative antigen influx into the mucosa. Permeabilities for fluorescein (332 Da; 100 µmol/L) and larger molecules such as fluorescein-labelled (FITC-) dextran (4 kDa; 200 µmol/L) were studied. Flux studies were conducted from the apical to the basolateral compartment in six-well plates at 37 °C with a glucose-enriched (10 mmol/L) HEPES-buffered Ringer. Samples were taken from the basolateral compartment every 15 min for one hour for fluorescein and every 30 min for two hours for FITC-dextran. Fluorescence was measured in a spectrophotometer (Tecan GmbH, Maennedorf, Switzerland), and permeability of the monolayers was calculated from flux over concentration difference.

### 4.3. Analysis of Epithelial Cell Death Mechanisms

To quantify cellular cytotoxicity the CyQUANT LDH Cytotoxicity Assay (Invitrogen, Carlsbad, CA, USA) was performed according to the manufacturer’s instructions. Untreated filters were used as the maximal LDH activity controls and lysed with the kit’s 10× Lysis Buffer for 45 min at 37 °C. Interferon gamma (IFN-γ; 500 U/mL) treated monolayers (basolateral, 72 h) served as positive control of cytotoxicity induction. Percentage of cytotoxicity in untreated or toxin and cytokine treated monolayers was determined. Thereby, spontaneous LDH activity corresponds to LDH activity of untreated cells.

### 4.4. Tight Junction Protein Expression Evaluation

For analysis of the TJ composition, toxin-treated human colonic organoid monolayers, as well as untreated cells were washed twice with ice-cold PBS and extracted with ice-cold whole cell lysis buffer (150 mmol/L NaCl, 10 mmol/L Tris buffer (pH 7.5), 0.5% Triton X-100, 1% SDS, and Complete Protease Inhibitor (Roche AG, Manheim, Germany)). Subsequent to lysis, cells were scraped carefully from the filters and incubated on ice for 1 h and vortexed every 10 min. Lysates were centrifuged for 30 min at 13,000 rpm at 4 °C. The supernatant was used for total protein quantification by Pierce BCA assay (Thermo Fisher Scientific, Waltham, MA, USA), according to the manufacturer’s instruction. Afterwards, protein samples (20 µg) were electrophoretically separated on 12.5% SDS polyacrylamide gels, transferred to a nitrocellulose membrane for 1 h and blocked with 1% PVP-40 (Polyvinylpyrrolidone; Sigma Aldrich, St. Louis, MO, USA) in TBST/0.05%Tween-20 (TBS-T) buffer for 30 min at room temperature. Following this, membranes were incubated overnight at 4 °C in primary antibodies against mouse (ms) claudin-1, ms claudin-2, rabbit (rb) claudin-3, ms claudin-4, ms claudin-5, rb claudin-7, rb claudin-12 (1:1000 Invitrogen, Carlsbad, CA, USA), rb tricellulin (1:2000; Invitrogen), rb occludin (1:1000; Proteintech Rosemont, IL, USA), and ms GAPDH as a loading control (1:10,000; Sigma Aldrich) on a shaker. On the next day, membranes were washed with TBS-T and incubated with appropriate secondary antibodies (peroxidase-conjugated goat anti-rabbit IgG and goat-anti-mouse IgG, Jackson ImmunoResearch, Ely, UK) at room temperature for 2 h. Proteins were then detected with SuperSignal West Pico PLUS Stable Peroxide Solution (Thermo Fisher Scientific) and the FUSION FX imaging system (Vilber Lourmat Deutschland GmbH, Eberhardzell, Germany). Protein bands were quantified via densitometric analysis of the Western blots by ImageJ software (Rasband, W. S., ImageJ, National Institute of Health (NIH), Bethesda, MD, USA).

### 4.5. Analysis of Subcellular Tight Junction Protein Localization via Confocal Laser-Scanning Microscopy and Super-Resolution STED Microscopy

Confocal laser-scanning microscopy (CLSM) or stimulated emission depletion (STED) microscopy was used to visualize TJ proteins and their subcellular localization in human colonic organoid monolayers. These monolayers on PCF filters were rinsed twice with PBS and fixed with 2% paraformaldehyde (PFA; Electron Microscopy Science, Hatfield, PA, USA) for 20 min. Afterwards, the cells were quenched with 25 mmol/L Glycine (Biomol GmbH, Hamburg, Germany), washed five times with PBS, and permeabilized for 10 min with 0.2% Triton X-100. Fixed and permeabilized cells were washed and incubated with blocking solution (5% goat serum (Gibco), 1% BSA (Sigma-Aldrich), 0.05% Tween-20, 0.01% Triton X-100) for 1 h at room temperature. Blocked cells were incubated with primary antibodies against claudin-1 (1:100; Invitrogen), claudin-2 (1:100; Invitrogen), claudin-4 (1:100; Invitrogen), occludin (1:100; Invitrogen), tricellulin (1:400; Invitrogen), and ZO-1 (1:100; BD Biosciences, Franklin Lakes, NJ, USA) overnight at 4 °C. Secondary antibodies were incubated for 2 h at room temperature. They were conjugated to Alexa Fluor 488 or 594 (1:500; Invitrogen) for CLSM and for STED Aberrior STAR RED and Aberrior STAR ORANGE (1:200; Abberior GmbH, Göttingen, Germany) antibodies were used. F-actin was stained using Phalloidin DY-647P1 (1:500; Dyomics GmbH, Jena, Germany) or Abberior STAR RED (1:100; Abberior GmbH). Nuclei were stained with DAPI (1:1000; Roche AG) only for CLSM. After incubation, the cells were washed twice with PBS and once with distilled water, then mounted on glass slides using either ProTaq Mount Fluor (Biocyc, Luckenwalde, Germany) or a mounting medium (Abberior GmbH) heated to 65 °C. For STED microscopy, STED-compatible cover slides (Carl Zeiss, Jena, Germany) were used. Confocal laser scanning (Zeiss LSM780, Jena, Germany) and STED (Abberior Facility Line, Abberior GmbH) microscopes were used to analyze the subcellular distribution of TJ proteins.

### 4.6. Flow Cytometry

Following stimulation with TcdB, cells were fixed with Fix/Perm solution (BD Biosciences), washed with PBS 0.5% bovine serum albumin and intracellularly labeled for 15 min at room temperature with antibodies against human cytokines diluted in Wash/Perm buffer (BD Biosciences). The following antibodies were used: mouse anti-IFN-γ (B27; BD Biosciences), mouse anti-IL-13 (Jes10-5A2.2; Miltenyi Biotec, Bergisch-Gladbach, Germany), mouse anti-IL-2 (5.344.111; BD Biosciences), human anti-TNF-α (cA2; Miltenyi Biotec), mouse anti-IL-1β (AS10; BD Biosciences), and rat anti-IL-6 (MQ2-13A5; Biolegend, San Diego, CA, USA). Following washing and resuspension with Wash/Perm buffer, data were acquired on the FACSCanto II system (BD Biosciences) and analyzed with FlowJo software version 10.8.1. (BD Biosciences). Epithelial cells were gated based on time with continuous flow rate, doublet discrimination and characteristic forward and side scatter properties. These cells were further subdivided into individual populations positive for the respective cytokines. Percentages of cytokine positive cells were calculated, averaged and log2-fold change was calculated for cells treated with 1 ng/mL and 10 ng/mL TcdB compared to untreated cells.

### 4.7. RNA-Seq Expression Analysis

RNA-sequencing (RNA-seq) together with bioinformatics pathway analysis was conducted in order to gain insight in cell signaling during exposure of intestinal epithelial cells to the clostridial toxins. We used another intestinal cell model, the HT-29/B6-GR/MR cell line [52], which was established in prior experimental series also for the pathway analysis via RNA-seq [41,53].

HT-29/B6-GR/MR cell monolayers were treated with 0.1 ng/mL TcdA or 0.1 ng/mL TcdB for 20 h and total RNA was isolated using the mirVana miRNA Isolation Kit (Ambion, Life Technologies, Carlsbad, CA, USA). RNA-seq was performed using the TrueSeq Stranded Total RNA method on a NovaSeq 6000 Sequencing System with quality scores of 80%. Data and meta-data were stored in the SODAR FAIR data management system. The reads from RNA-Seq were trimmed with the trimadap program and mapped against the human genome GRCh38 patch release 7 and sorted using the STAR aligner version 2.7.8a [54,55]. Read counts were calculated using the coordinates from the GENCODE v25 annotation. Raw counts were obtained directly from the Star program. Quality control was performed using the programs FastQC [56], dupradar [57], rna_seqc [58] and summarized using MultiQC [59]. The Bioconductor package DESeq2 was used to quantify the differential expression of genes between two conditions in form of log2-fold changes with their corresponding *p*-values; *p*-values were adjusted to control for false discovery rate (FDR) using the Benjamini–Hochberg procedure. Pathway analysis was performed with ingenuity pathway analysis software (IPA, Qiagen Silicon Valley, Redwood, CA, USA) to identify signaling patterns or upstream regulators that could be responsible for the changes in barrier function after toxin exposure likewise performed previously [52]. For gene set enrichment analysis, the algorithm CERNO as implemented in the R package tmod was used [60]. KEGG, REACTOME, GO BP and Hallmark gene sets were derived from the MSigDB [61]. All QC, mapping, and analysis steps were performed using the seasnap pipeline available online from https://github.com/bihealth/seasnap-pipeline (accessed on 1 May 2023). Fastq files containing the unprocessed raw reads from sequencing and a raw counts matrix table are publicly available at Gene Expression Omnibus (GEO) archive under National Centre for Biotechnology Information (NCBI) website with GEO accession ID GSE232704.

### 4.8. Statistics

Data are expressed as mean values ± standard error of the mean (SEM). For statistical analysis GraphPad Prism software was used (GraphPad Software version 7, San Diego, CA, USA). *p*-values were calculated using one-way ANOVA with Bonferroni–Holm adjustment. *p* < 0.05 was considered statistically significant.

## Data Availability

The raw data supporting the findings of this manuscript will be made available by the corresponding author, R.B., or the first author, M.S., to any researcher upon reasonable request. RNA-seq datasets were deposited at GEO accession ID GSE232704. Available online: https://www.ncbi.nlm.nih.gov/geo/query/acc.cgi?acc=GSE232704 (accessed on 1 June 2023).

## Supplementary Materials

Pathway analysis from RNA-sequencing. Upstream regulators after clostridial toxin exposure. The following supporting information can be downloaded at: https://www.mdpi.com/article/10.3390/toxins15110643/s1. The entire list of activated and inactivated pathways in Upstream regulator analysis (IPA software). To expand the list with the affected pathways, chose the filter button in Molecule Type tab and select All. Caption Table S1: Upstream regulators after TcdA exposure. An upstream regulator analysis was performed with Ingenuity Pathway Analysis (IPA, Qiagen Silicon Valley, Redwood City, CA, USA) from toxin-exposed intestinal HT-29/B6-GR/MR epithelial cell monolayers. The basis for the bioinformatics analysis were RNA-seq datasets for comparison of untreated controls and TcdA-treated cells. The expression log ratio, predicted activation state, activation Z-score, *p*-value of overlap, and target molecules of different upstream regulators activated by TcdA 20 h after treatment were shown in the table. In this presentation of the table, cytokines as upstream regulators were shown, including barrier-affecting cytokines (bold) such as tumor necrosis factor-α (TNF-α), interferon-γ (INF-γ) and interleukin-1β (IL-1β) as activated pathway in the Predicted Activation State column. Additionally, upstream regulator TNF superfamily member 11 (TNFSF11) was found to be in an activated state, which targets the small GTPase ras homolog gene family, member B (RHOB). Further cytokines such as interleukin (IL)-13, IL-2, IL-4, IL-17A, Oncostatin-M (OSM), Endothelin-1 (EDN1) or colony stimulating factor 1 (CSF1) were also activated although with a lower activation z-score. IL-13 is an important cytokine in regulation of epithelial physiology in the colon. Remarkably, small GTPases such as RHOA and RHOB and Ezrin (EZR) were often listed in the expanded Table S1 as Target Molecules (regulators of cytoskeleton and epithelial organization). Caption Table S2: Upstream regulators after TcdB exposure. Upstream regulator analysis with IPA from toxin-exposed intestinal HT-29/B6-GR/MR epithelial cell monolayers. RNA-seq datasets for comparison of untreated controls and TcdB-treated cells were used for Upstream regulator analysis as explained above. In this Table S2, also the barrier-affecting cytokines IL-13, TNF-α, INF-γ and IL-1β show up among other cytokine signaling pathways. In the expanded list of the table (All Molecule Types), substances show up with inactivated Predicted Activation State and negative values in Activation z-score, such as wortmannin or sirolimus. This could be an indication of a counter-regulation of the target genes, so that the toxic effect of TcdB can be treated by a pharmaceutical agent. Caption Figure S1: Pathway analysis from RNA-sequencing in toxin-exposed intestinal HT-29/B6-GR/MR epithelial cells. Panel plot of gene set enrichment results for the TcdA and TcdB compared to controls. Only gene sets significant at FDR < 0.05 are shown. Furthermore, only gene sets with AUC > 0.55 and FDR < 0.01 in at least one comparison are shown. Each row corresponds to one gene set. Each column corresponds to one comparison. The length of the bars corresponds to the effect size (AUC, area under the curve). Color strength corresponds to FDR (*p*-value adjusted for multiple testing using the Benjamini-Hochberg procedure). Colored fragments indicate the proportion of genes which are significantly up- (red) or down- (blue) regulated in the gene set. Grey fragments indicate the proportion of genes which are not significantly regulated in the gene set.
