# Supplementary material for: A Colonic Organoid Model Challenged with the Large Toxins of Clostridioides difficile TcdA and TcdB Exhibit Deregulated Tight Junction Proteins
Source: Toxins (Basel). 2023 Nov 4;15(11):643. doi: 10.3390/toxins15110643 (PMC10674794; doi:10.3390/toxins15110643)
Supplement: Supplementary file 1 [file toxins-15-00643-s001.zip › toxins-2674492-supplementary.pdf]

toxins

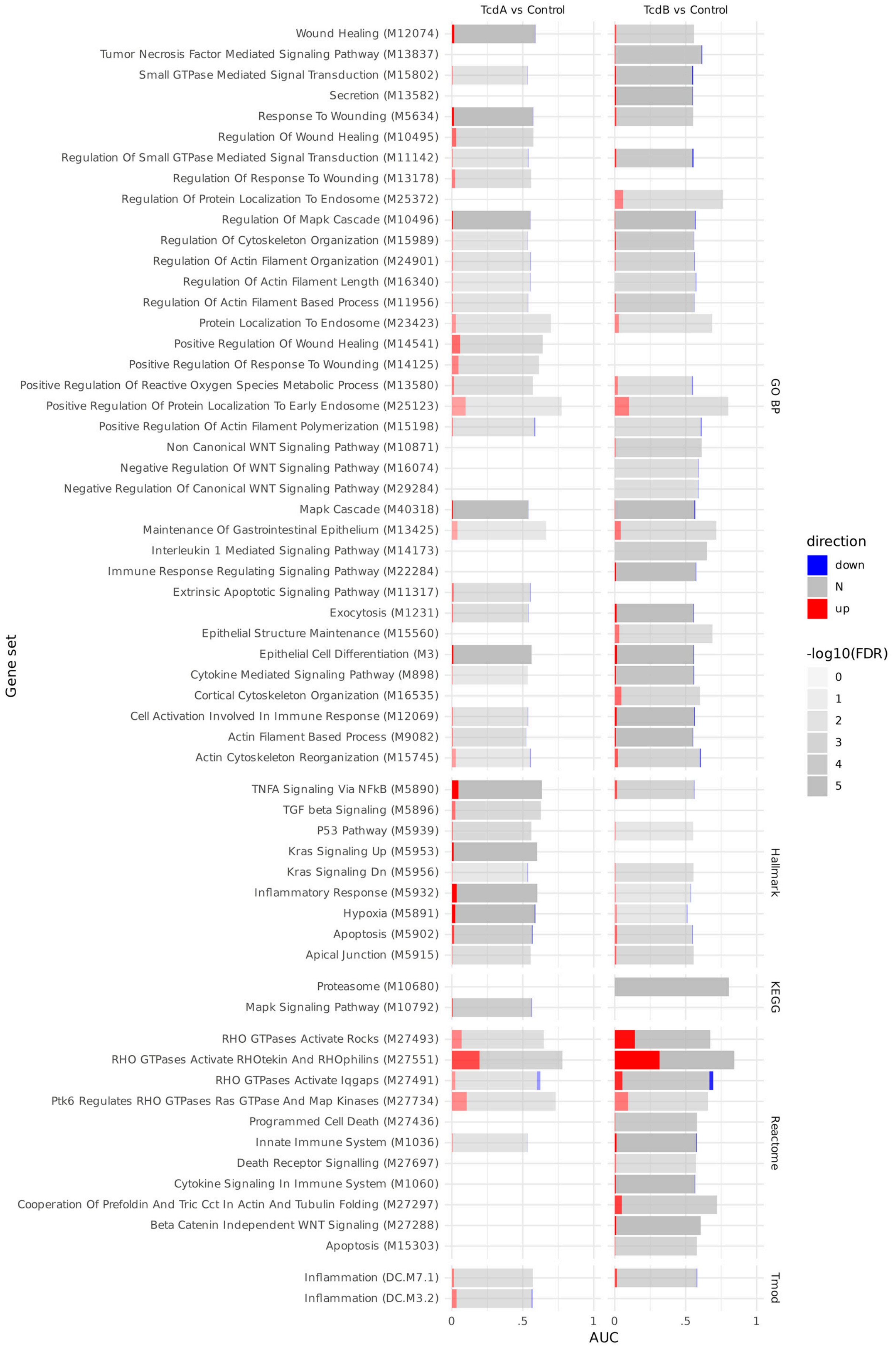
TcdA vs Control
TcdB vs Control
Wound Healing (M12074)
Tumor Necrosis Factor Mediated Signaling Pathway (M13837)
Small GTPase Mediated Signal Transduction (M15802)
Secretion (M13582)
Response To Wounding (M5634)
Regulation Of Wound Healing (M10495)
Regulation Of Small GTPase Mediated Signal Transduction (M11142)
Regulation Of Response To Wounding (M13178)
Regulation Of Protein Localization To Endosome (M25372)
Regulation Of Mapk Cascade (M10496)
Regulation Of Cytoskeleton Organization (M15989)
Regulation Of Actin Filament Organization (M24901)
Regulation Of Actin Filament Length (M16340)
Regulation Of Actin Filament Based Process (M11956)
Protein Localization To Endosome (M23423)
Positive Regulation Of Wound Healing (M14541)
Positive Regulation Of Response To Wounding (M14125)
Positive Regulation Of Reactive Oxygen Species Metabolic Process (M13580)
Positive Regulation Of Protein Localization To Early Endosome (M25123)
Positive Regulation Of Actin Filament Polymerization (M15198)
Non Canonical WNT Signaling Pathway (M10871)
Negative Regulation Of WNT Signaling Pathway (M16074)
Negative Regulation Of Canonical WNT Signaling Pathway (M29284)
Mapk Cascade (M40318)
Maintenance Of Gastrointestinal Epithelium (M13425)
Interleukin 1 Mediated Signaling Pathway (M14173)
Immune Response Regulating Signaling Pathway (M22284)
Extrinsic Apoptotic Signaling Pathway (M11317)
Exocytosis (M1231)
Epithelial Structure Maintenance (M15560)
Epithelial Cell Differentiation (M3)
Cytokine Mediated Signaling Pathway (M898)
Cortical Cytoskeleton Organization (M16535)
Cell Activation Involved In Immune Response (M12069)
Actin Filament Based Process (M9082)
Actin Cytoskeleton Reorganization (M15745)
GO BP
TNFA Signaling Via NFkB (M5890)
TGF beta Signaling (M5896)
P53 Pathway (M5939)
Kras Signaling Up (M5953)
Kras Signaling Dn (M5956)
Inflammatory Response (M5932)
Hypoxia (M5891)
Apoptosis (M5902)
Apical Junction (M5915)
Hallmark
Proteasome (M10680)
Mapk Signaling Pathway (M10792)
KEGG
RHO GTPases Activate Rocks (M27493)
RHO GTPases Activate RHOtekin And RHOphilins (M27551)
RHO GTPases Activate Iqgaps (M27491)
Ptk6 Regulates RHO GTPases Ras GTPase And Map Kinases (M27734)
Programmed Cell Death (M27436)
Innate Immune System (M1036)
Death Receptor Signalling (M27697)
Cytokine Signaling In Immune System (M1060)
Cooperation Of Prefoldin And Tric Cct In Actin And Tubulin Folding (M27297)
Beta Catenin Independent WNT Signaling (M27288)
Apoptosis (M15303)
Reactome
Inflammation (DC.M7.1)
Inflammation (DC.M3.2)
Tmod
Gene set
0
.5
1
0
.5
1
AUC
direction
down
N
up
-log10(FDR)
0
1
2
3
4
5

Figure S1: Pathway analysis from RNA-sequencing in toxin-exposed intestinal HT−29/B6−GR/MR epithelial cells. Panel plot of gene set enrichment results for the TcdA and TcdB compared to controls. Only gene sets significant at FDR < 0.05 are shown. Furthermore, only gene sets with AUC > 0.55 and FDR < 0.01 in at least one comparison are shown. Each row corresponds to one gene set. Each column corresponds to one comparison. The length of the bars corresponds to the effect size (AUC, area under the curve). Color strength corresponds to FDR (*p*−value adjusted for multiple testing using the Benjamini−Hochberg procedure). Colored fragments indicate the proportion of genes which are significantly up− (red) or down− (blue) regulated in the gene set. Grey fragments indicate the proportion of genes which are not significantly regulated in the gene set.
